# Genome-Wide Characterization and Expression Analysis of the Germin-Like Protein Family in Rice and Arabidopsis

**DOI:** 10.3390/ijms17101622

**Published:** 2016-09-23

**Authors:** Lu Li, Xihui Xu, Chen Chen, Zhenguo Shen

**Affiliations:** College of Life Sciences, Nanjing Agricultural University, Nanjing 210095, China; 2014216005@njau.edu.cn (L.L.); xuxihui@njau.edu.cn (X.X.); zgshen@njau.edu.cn (Z.S.)

**Keywords:** *GLPs*, tandem duplication, phylogenetic analysis, expression pattern, cell wall, SOD activity

## Abstract

Previous studies have shown that germin-like proteins (GLPs) are present ubiquitously in rice and Arabidopsis. However, the understanding regarding their role in development and abiotic/biotic stress resistance remains limited. In the present study, we report genome-wide identification, characterisation, subcellular localization, enzyme activity, and expression analysis of the *GLP* gene family in rice and Arabidopsis to study their functions. In total, 43 and 32 *GLPs* in the rice and Arabidopsis genome were identified based on a systematic analysis, respectively. The *GLP* genes were clustered into six clades based on phylogenetic analysis, and many stress and developmental-related *cis*-elements were detected in promoters of *GLP* genes. In addition, subcellular location and superoxide dismutase (SOD) analysis demonstrated that the random selected *OsGLP* genes on chromosomes 8 and 4 of rice were expressed in the cell wall with SOD activity. Overall, our results showed that tandem duplication events, especially the clusters of tandem duplication genes on chromosome 8 in rice, play a major role in expansion of the *GLP* family and thus increase our understanding of the role of the *GLP* family in abiotic/biotic stress and development.

## 1. Introduction

Germin and germin-like proteins (GLPs) were first discovered in wheat seeds as specific markers of germination [1,2], after which they were widely found in monocotyledons, dicotyledons, and gymnosperms [3]. The germin family belongs to the functionally diverse cupin superfamily, and generally codes two exons, and contains a “cupin” (PF00190) at its C-terminus [4]. It is a challenging work to classify germins and GLPs, due to their high conserved sequence and the similarity of structural characteristics [5]. In general, the “true germins” belong to a well-conserved homogeneous group and are almost uniquely found within cereal plant species [6,7,8], while GLP proteins belong to a heterogeneous group and have a wider taxonomic coverage in plants [2,9].

Most GLPs are reported to have enzyme activities only in the polymeric form [10,11,12], but one GLP protein in *Capsicum chinense* showed superoxide dismutase (SOD) activity without forming polymers [13]. In barley, six germin proteins that each combines a single manganese-ion, form an extremely stable hexamer protein structure [9]. In addition, GLPs have been reported to possess other enzyme activities, such as functioning as an auxin receptor [14], oxalate oxidase (OXO) activity [12], as well as polyphenol oxidase [11] and serine protease inhibitors [15].

*GLP* genes are expressed in all types of organs including leaves, cotyledons, stems, roots, embryos, flowers, and seeds, and are involved in developmental processes [3,16]. The overexpression of *GLP* genes in Arabidopsis and rice influenced the normal growth and development of plants [17,18]. Besides, *GLP* genes have different spatial and temporal expression characteristics in a variety of plants, which could affect the enzyme activities [3]. For example, *GLP* genes are expressed in the apoplast and cell wall of embryonic cells compared with OXO activity during germination, and those genes were considered to be plant cell defenders [19]. The quaternary structure of GLPs is highly resistant to heat, extreme pH, proteases, and sodium dodecyl sulfonate (SDS) [10,20]. High *GLP* gene expressions have been observed under different abiotic stresses, such as salt stress [16], drought stress [16], heavy metal stress [19,21] and wound stress [16]. The *GLP* genes are also expressed under many biotic stresses, such as in the presence of fugal pathogens [10,22], bacteria [23], and viruses [13]. Expressions of *GLP* genes have also been shown to be increased within the plant cell wall after infection, and the mechanism by which GLPs influence plant defence is likely related to ROS (reactive oxygen species) production and formation of an “oxidative burst” response [23,24]. Recent studies revealed a close connection between both *GLP* gene clusters and disease resistance phenotypes [25,26]. However, the functions of many *GLP* genes are still largely unknown, and the response of *GLP* gene tandem clusters or single *GLP* genes to biotic/abiotic stresses needs to be identified.

Although the functions of some *GLP* genes have been characterised in barley [27], wheat [28], soybean [3], and moss [29], a comparison of the *GLP* family between monocotyledon and dicotyledon has never been performed. In this study, members of the *GLP* family in rice and Arabidopsis were reanalysed based on complete genome sequences and annotation. We proposed nomenclature, provided chromosomal distribution, identified tandem duplications, and performed phylogenetic analyses of *GLP* genes in Arabidopsis and rice. The expressions of *GLP* genes during development and under biotic/abiotic stress conditions were evaluated based on bioinformatics analysis, and the “hotspot” genes for biotic/abiotic stress were identified. In addition, quantitative real time PCR (qRT-PCR) analyses of rice *GLP* genes under four different abiotic stresses were performed and the subcellular localisation of three GLPs in rice were analysed. Our study will provide a reference for further functional analyses of members of the *GLP* family in rice and Arabidopsis.

## 2. Results and Discussion

### 2.1. Identification and Nomenclature of GLP Genes in Arabidopsis and Rice Genomes

A total of 32 distinct chromosomal loci encoding for 37 *GLP* genes in Arabidopsis and 43 chromosomal loci encoding for 48 *GLP* genes in rice were identified (Table S1). Previous studies reported 29 *AtGLP* genes in Arabidopsis [18] and 41 *OsGLP* genes in rice [25]. We found three new *GLP* genes (AT1G74820, AT5G39100, AT5G61750) in Arabidopsis and two new *GLP* genes (Os01g14670, Os03g58990) in rice. Manosalva et al. divided *GLP* genes in rice into six groups (from *OsGER1* to *OsGER6*) according to the barley nomenclature [25], while *GLP* genes in Arabidopsis have not been systematically denominated. To maintain uniformity and avoid ambiguity, we proposed new nomenclature for *GLP* family members in this study (Table S1). We numbered the *GLP* genes according to their 1–5 chromosomal location for Arabidopsis, and 1–12 chromosomal location for rice, and from top to bottom. Details of each *GLP* member, including the locus ID, open reading frame length, protein length, and chromosomal location of all *GLP* genes, are shown in Table S1.

### 2.2. Chromosomal Distribution of GLP Genes

**Figure 1:**
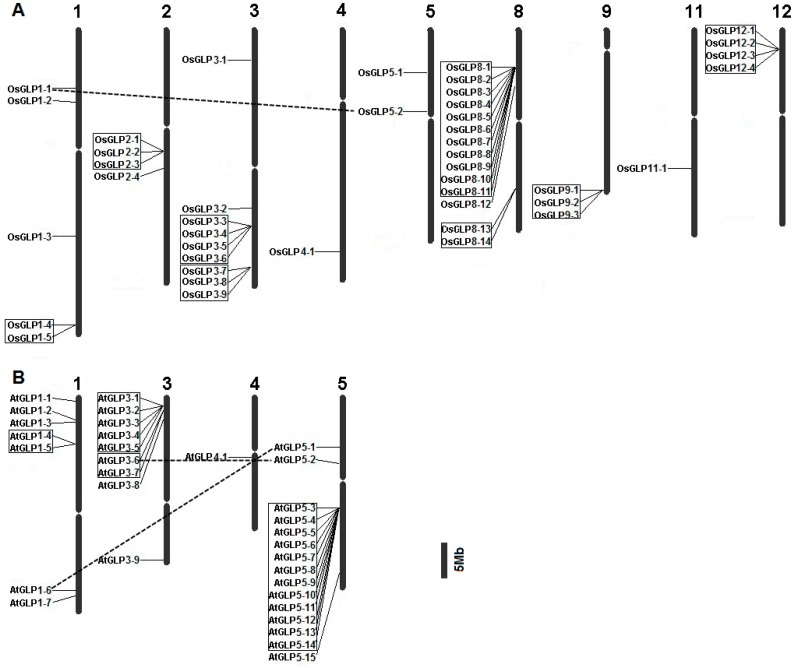
Chromosomal distribution of rice (**A**) and Arabidopsis (**B**) germin-like protein (GLP) genes. The chromosome numbers are shown at the top of the chromosomes and the centromeric regions are indicated by ellipses. Tandem duplicated genes are shown in boxes, and the segmentally duplicated genes are connected by dashed lines. The exact position of each *GLP* gene on rice and Arabidopsis chromosome pseudo-molecules is given in Table S1.

To determine the chromosomal distribution of *GLP* genes in rice and Arabidopsis, chromosomal maps were constructed (Figure 1). In rice, the *OsGLP* genes are distributed on nine of 12 chromosomes, excluding chromosomes 6, 7, and 10 (Figure 1A). Chromosome 8 encoded the highest number (14 of 43, 32.6%) of *OsGLP* genes, followed by chromosome 3 (9 of 43, 20.9%) and chromosome 1 (5 of 43, 11.6%). Tandem duplications were observed among 32 genes forming eight clusters on chromosomes 1, 2, 3, 8, 9, and 12. Maximum tandem duplicated genes were found on chromosome 8 (11 members), which also harboured another cluster with two *OsGLP* genes. In Arabidopsis, chromosome 5 encoded the maximum 15 *AtGLP* genes (Figure 1B), and tandem duplications were also observed among 21 genes forming four clusters. The largest cluster localised on chromosome 5 contains 12 *GLP* genes presenting in tandem at a single locus. Besides, two pairs of *GLP* genes were found to be segmentally duplicated in Arabidopsis, whereas only one pair of *GLP* genes was segmentally duplicated in rice. Tandem duplications of genes from rice oxalate oxidase cupin subclasses have also been reported [7]. Tandem duplication was common among chromosomes of rice and Arabidopsis, which may contribute to the plant evolution of the *GLP* family [30] and function under “abiotic and biotic stress”. For both Arabidopsis and rice, the number of *GLP* genes present in tandem is much larger than those located on the segmentally duplicated region. Thus, tandem duplications appear to play an important role in expansion of the *GLP* family in rice and Arabidopsis.

### 2.3. Phylogeny and Structure Analysis of GLP genes

**Figure 2:**
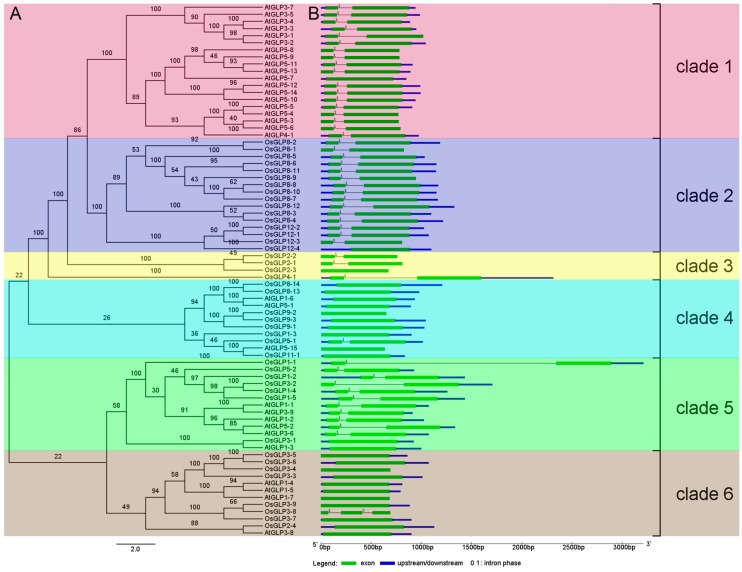
Phylogenetic relationships (**A**) and exon/intron structure (**B**) of *GLP* genes of rice and Arabidopsis. Multiple sequence alignment of nucleotide sequences was performed using the MAFFT program and a phylogenetic tree was generated using Bayesian inference (BI). Posterior probabilities (scaled to 100) are showed on the branch. The gene structure schematic diagrams were drawn using the Gene Structure Display Server (GSDS); (http://gsds1.cbi.pku.edu.cn/). The exons, introns and upstream/downstream sequences are indicated by green rectangle, black lines and blue rectangle, respectively. The length of the rectangles and lines are scaled based on the length of the gene.

To elucidate the evolutionary significance of *GLP* genes across Arabidopsis and rice, phylogenetic analysis was performed using conserved regions of *OsGLP* and *AtGLP* sequences (Figure 2). The rice genome encodes a significantly higher number of *GLP* genes (43 genes) compared to Arabidopsis (32 genes), indicating a more rapid evolutionary rate in rice than Arabidopsis. According to the phylogenetic tree, all *GLP* genes of Arabidopsis and rice could be divided into six major clades. All *GLP* genes from clade 1 belong to Arabidopsis, which are mainly comprised of two tandem clusters from chromosomes 3 and 5. *GLP* genes from two tandem clusters on rice chromosomes 8 and 12 constitute clade 2. Clade 3 is comprised of *GLP* genes in rice, with one tandem cluster from chromosome 2 and one *GLP* gene from chromosome 4 (*OsGLP4-1*). Besides, other *GLP* genes from Arabidopsis and rice were clustered together (clades 4, 5, and 6). These results indicated that *GLP* genes existed before the divergence of monocots and dicots, and some *GLP* genes expanded independently in a species-specific manner. This species-specific expansion pattern has been reported in other gene families, such as the VQ motif-containing protein family [31] and zinc finger-homeodomain gene family [32].

Many studies proved that gene structural diversity is a possible mechanism for the evolution of multi-gene families [33,34]. To increase the understanding of the structural diversity of *GLP* genes, we compared the exon/intron organisation in the coding sequences of individual *GLP* genes in rice and Arabidopsis (Figure 2B). In general, we found that most closely related members in the same clade shared a similar exon/intron structure in terms of intron number and exon length. For example, no intron is present in *GLP* members of clade 4, except *OsGLP5-1*, and clade 6 contains no introns except *OsGLP3-8*. All *GLP* genes in clades 1, 2, and 3 contained one intron, excluding *AtGLP5-7*, *OsGLP12-4*, and *OsGLP2-3*, which do not possess an intron.

### 2.4. Differential Expression of GLP Genes during Development

**Figure 3:**
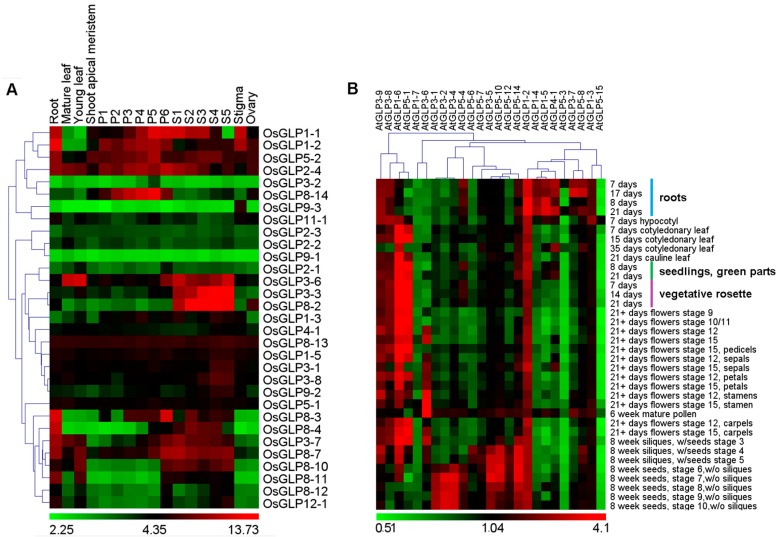
Expression patterns of rice and Arabidopsis *GLP* genes in various tissues/organs and developmental stages. Hierarchical clustering analysis of 31 *OsGLP* genes (**A**) and 25 *AtGLP* genes (**B**) represented on an Affymetrix genome array is shown. Reproductive development comprising six stages of panicle (P1 (0–3 cm), P2 (3–5 cm), P3 (5–10 cm), P4 (10–15 cm), P5 (15–22 cm), and P6 (22–30 cm)) and five stages of seed tagged from day of pollination (S1 (0–2 DAP), S2 (3–4 DAP), S3 (5–10 DAP), S4 (11–20 DAP), and S5 (21–29 DAP)) development. For clustering we used average log signal values (log10 for Arabidopsis and log2 for rice) for three biological replicates of each sample after normalisation of raw data (Tables S2 and S3). The colour scale for log signal values is shown at the bottom.

To gain insight into the possible function of *GLP* genes during development, we analysed the expression pattern of *OsGLP* and *AtGLP* genes in various tissues/organs and developmental stages using microarray data (Tables S2 and S3). For rice *GLP* genes, Affymetrix GeneChip rice genome arrays (GSE6893 and GSE7951) were used, and 31 *OsGLP* genes were represented (Figure 3A).

In general, some *OsGLP* genes only expressed in certain tissues or developmental stages while others showed high expressions during all developmental stages and in different tissues. For example, the expressions of *OsGLP3-3* and *OsGLP8-2* were restricted to seed development stages (S1–S5). *OsGLP8-14* was preferentially expressed during panicle development stages P1 to P6, while the expression of *OsGLP9-3* was restricted to stigma. The expressions of *OsGLP3-6*, *OsGLP3-7* and *OsGLP8-10* were exhibited preferentially in vegetative tissues and the stages of panicle and seed development. These genes may perform specific roles in these tissues/organs or developmental stages. It should be noted that some *GLP* genes including *OsGLP5-2*, *OsGLP2-4*, and *OsGLP8-13*, were expressed at high levels in almost all developmental stages, suggestive of their broad role in plant development. Similar analysis was performed for *AtGLP* genes (Figure 3B). Additionally, some of the *GLP* genes, such as *AtGLP3-9*, *AtGLP3-8*, and *AtGLP1-2*, were highly expressed during all developmental stages, while the expressions of *AtGLP5-3*, *AtGLP5-15*, and *AtGLP1-7* were lower during almost all developmental stages. The high expressions of *AtGLP3-5* and *AtGLP5-10* in stages 8, 9, and 10 of seed development supported their role in seed development. Interestingly, a close relationship between gene expression profiles and their clustering in the chromosomes was found. For example, the tandem duplicated gene such as *OsGLP2-1* to *OsGLP2-3* from chromosome 2 expressed at low level, while most *OsGLP* genes from one cluster of chromosome 8 such as *OsGLP8-2*, *OsGLP8-3*, *OsGLP8-4*, *OsGLP8-7*, *OsGLP8-10*, and *OsGLP8-11* showed high expression in seed. The *AtGLP* cluster from chromosome 1 showed high expression in root, while most of the *AtGLP* genes from one cluster of chromosome 3 expressed mainly in seed. In addition, the segmentally duplicated genes *AtGLP1-6* and *AtGLP5-1* showed similar expression during the studied Arabidopsis developmental stage.

Moreover, massively parallel signature sequencing (MPSS) data was analysed to quantify the expression of *GLP* genes in rice (Table S4) and Arabidopsis (Table S5). Signature tags were found for 22 *GLP* genes in rice, while there were only 15 identified in Arabidopsis. The expression profiles of *GLP* genes obtained from MPSS data agreed largely with microarray data.

### 2.5. Differential Expression of GLP Genes during Abiotic Stress

**Figure 4:**
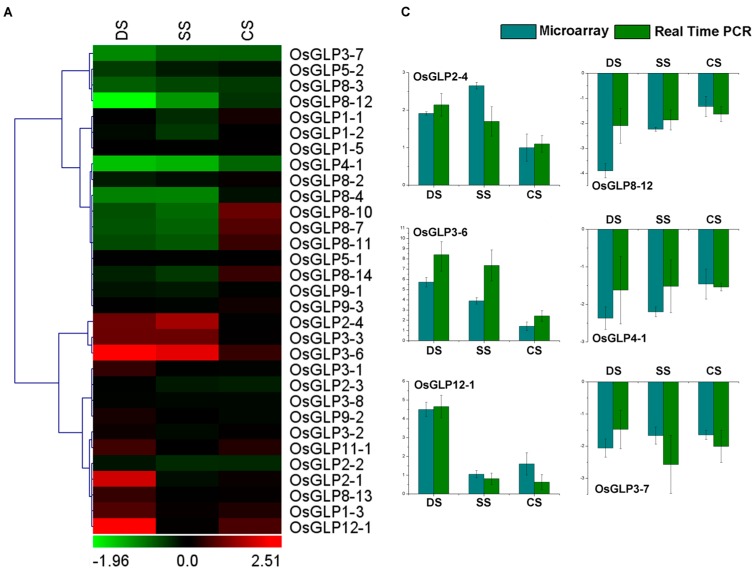

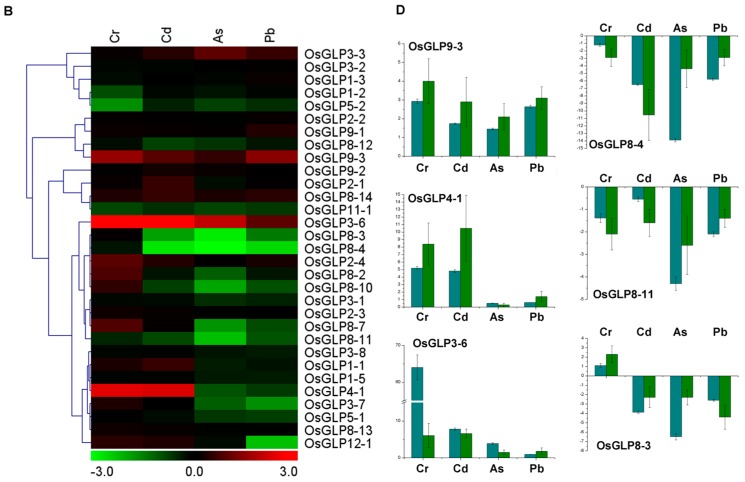
Differential expressions of rice *GLP* genes in response to various abiotic stresses. Hierarchical clustering of rice *GLP* genes showing significantly different expressions under at least one abiotic stress condition (**A**) or heavy metals (**B**) is shown. The log2 fold change of *GLP* gene expressions (Table S6) in treated samples compared with mock-treated control sample was used for clustering. The colour scale for log2 fold change values is shown at the bottom; (**C**,**D**) Real-time PCR analysis of random selected genes to validate their differential expression during various abiotic stress conditions. The mRNA levels for each gene in different tissue samples were calculated relative to its expression in control seedlings. The green colour represents downregulation, black signifies no change in expression, and red shows upregulation. The error bars represent standard deviation. DS, desiccation stress; SS, salt stress; CS, cold stress.

**Figure 5:**
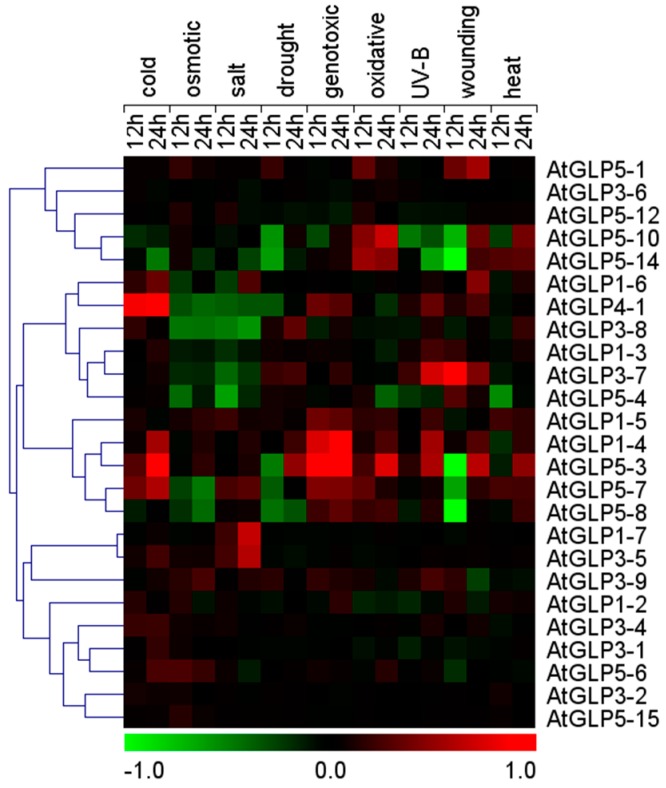
Microarray-based expression profile of Arabidopsis *GLP* genes under various abiotic stress conditions. Heat maps show the log10 fold changes of Arabidopsis *GLP* gene expressions in root tissues under different abiotic stress conditions such as salt, drought, osmotic, cold, heat, oxidative, genotoxic, wounding, and UV/B stress. Microarray data (Table S7) was obtained for different time points and stresses *viz*. 12 and 24 h for root tissues and analysed with respect to the control. Relative signal values are represented by the colour bar shown at the bottom of heat map; green colour represents downregulation, black signifies no change in expression, and red shows up-regulation.

We analysed the differential expression of *GLP* genes of rice seedlings under different abiotic stresses (desiccation, salt, cold, and heavy metals) with microarray data of GSE6901 [35] and GSE25206 [36] (Figure 4; Table S6). *OsGLP3-7*, *OsGLP4-1*, and *OsGLP8-12* were down-regulated under desiccation, salt and cold stress, while *OsGLP3-6* was up-regulated during these three stresses (Figure 4A). *OsGLP8-4*, *OsGLP8-10*, *OsGLP8-7*, and *OsGLP8-11* were down-regulated under desiccation and salt stress, but up-regulated or unchanged under cold stress. Similarly, *OsGLP2-4*, *OsGLP3-3*, and *OsGLP3-6* were up-regulated under desiccation and salt stress and nearly unchanged under cold stress. These results showed that cold stress did not affect the expression of *OsGLP* genes significantly. Besides, 11 *OsGLP* genes were differentially expressed by more than 2-fold under at least one of heavy metal stresses (Figure 4B). Overall, the expressions of the *OsGLP* genes in the largest cluster on chromosome 8 and that of *OsGLP4-1* on chromosome 4, were significantly regulated under various abiotic stresses (desiccation, salt, cold, and heavy metals) in rice. The differential expressions of representative *GLP* genes was also confirmed by qRT-PCR analysis (Figure 4C,D). Although the accurate fold changes of some genes obtained by microarray or qRT-PCR were slightly different, the variation tendencies of all the examined genes were identical. These results indicated that minimal variation in the expression data and high consistency between the results were obtained using these two techniques.Similarly, Arabidopsis microarray data in response to different abiotic stresses (cold, osmotic stress, salt, drought, genotoxic, oxidative, UV-B, wound, and heat) in root was retrieved from AtGenExpress (Figure 5, Table S7). Seven members in the tandem cluster of chromosome 5 might be involved in response to abiotic stresses (*AtGLP5-3*, *AtGLP5-4*, *AtGLP5-7*, *AtGLP5-8*, *AtGLP5-6*, *AtGLP5-10*, and *AtGLP5-14*), which were differently regulated under various abiotic stresses. Besides, the genes in tandem cluster on chromosome 1 (*AtGLP1-4* and *AtGLP1-5*) were up-regulated under certain abiotic stress. *AtGLP4-1* was up-regulated under cold stress, but down-regulated under osmotic and salt stresses. *AtGLP3-7* and *AtGLP3-8* were also down-regulated under osmotic and salt stresses, and *AtGLP3-7* was up-regulated under UV-B and wound stresses.

Some *GLP* genes have been implicated in various abiotic stress responses in plants [3,16,21]. For example, different expressions of *GLP* genes in response to salt in barley [37], cold in Arabidopsis [38], and heavy metals in rice [39] have been reported. It has been shown that GLPs possess SOD activity, this generates H_2_O_2_, which plays an important role in defending against various stresses [40]. For example, the overexpression of rice germin-like protein1 in tobacco hyper-accumulates H_2_O_2_ and reinforces the cell wall components, and consequently increased tolerance against biotic and abiotic stresses [41]. Some GLPs involved in antioxidant defence and detoxification were identified as Cu-IMAC-binding proteins [39], and Cu stress could decrease the expression of certain *GLP* genes, such as a *GLP* subfamily with a three member precursor in the Cu-tolerant plant *Elsholtzia splendens* [42]. The present study showed that the larger cluster of *OsGLP* genes on chromosome 8 and the single *OsGLP* gene on chromosome 4 in rice may be involved in abiotic stresses, similar to the cluster of *AtGLP* genes on chromosome 5. Arabidopsis has lost large amounts of sequence through deletion [43], while the tandem duplicates expanded in response to environmental stresses [44]. These “hotspot” *OsGLP* genes related to abiotic stress may be kept by Arabidopsis during natural selection. These genes have also been shown to be involved to biotic stress, which will be discussed below. 

### 2.6. Differential Expression of GLP Genes during Biotic Stress

**Figure 6:**
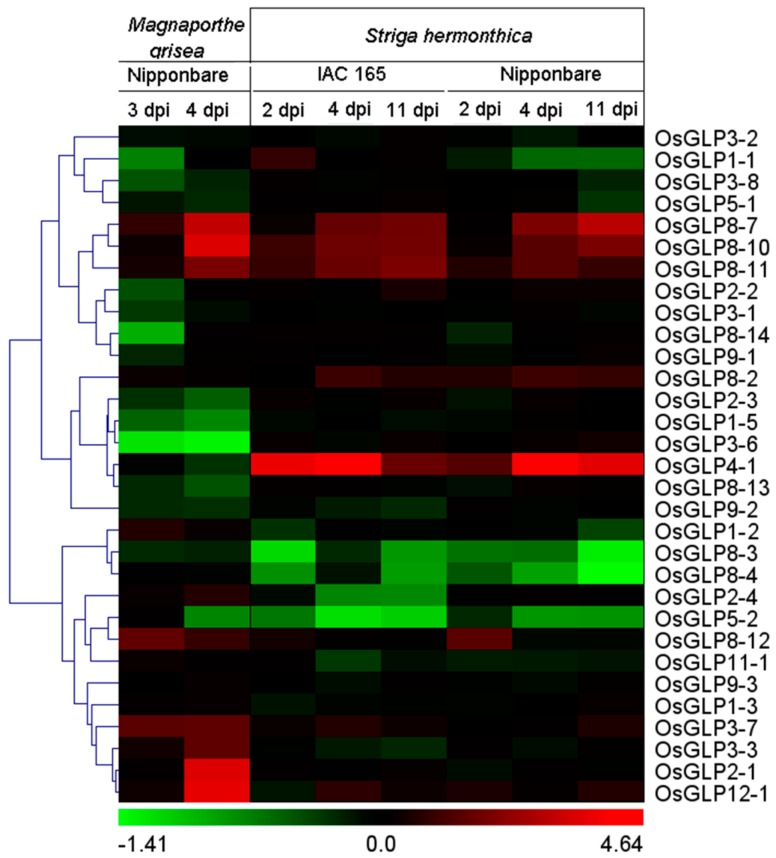
Differential expressions of rice *GLP* genes in response to various biotic stress conditions. Hierarchical clustering of *GLP* genes showing significant differential expressions in at least one condition is shown. The log2 fold change of *GLP* gene expressions (Table S8) in treated sample compared with a mock-treated control sample was used for clustering. The colour scale for log2 fold change values is shown at the bottom. The green colour represents downregulation, black signifies no change in expression, and red shows upregulation. Dpi, days of post-inoculation.

It has been shown that the expression of certain *GLP* genes is increased after infection with pathogens, feed of insects, or chemical application, indicating that GLPs may be involved in plant defence responses [25,27,45]. Rice blast is one of the most serious and widespread diseases caused by *Magnaporthe grisea* [25], and *Striga hermonthica* is a hemiparasitic weed that can infect cereals [46]. To study the effect of biotic stresses on the expressions of *GLP* genes in rice, the *OsGLP* gene responses to *M. grise* and two varieties of *S. hermonthica* (Nipponbare and IAC165) were analysed using GSE7256 and GSE10373 respectively [46,47]. A total of nine *GLP* genes were differentially expressed under *M. grisea* infection (Figure 6, Table S8). To be specific, four *OsGLP* genes on chromosome 8 (*OsGLP8-7*, *OsGLP8-10*, *OsGLP8-11*, and *OsGLP8-12*) were up-regulated. Besides, *OsGLP2-1*, *OsGLP3-3*, *OsGLP3-7*, and *OsGLP12-1* were up-regulated, and *OsGLP3-6* was down-regulated. As regards *S. hermonthica* infection, 8 *GLP* genes were differentially expressed by more than 2-fold under infection with the Nipponbare variety, while the expression of 6 genes changed after exposure to the IAC165 variety. Among them, the expression of three *OsGLP* genes on chromosome 8 (*OsGLP8-7*, *OsGLP8-10* and *OsGLP8-11*) and *OsGLP4-1* were significantly up-regulated, while *OsGLP8-3* was down-regulated in response to both two varieties. These results indicated that the tandem duplicated *OsGLP* genes on chromosome 8 might be involved in disease resistance. Previous studies have reported that these genes provide quantitative disease resistance as a quantitative trait loci (QTL) [25]. What is noteworthy is that the expression of *OsGLP4-1* was significant induced under *S. hermonthica* infection but not regulated under *M. grisea* challenging. Interestingly, both the expressions of *OsGLP1-5* and *OsGLP5-2*, located in segmentally duplicated regions, were slightly down-regulated under *M. grisea* infection*.*

**Figure 7:**
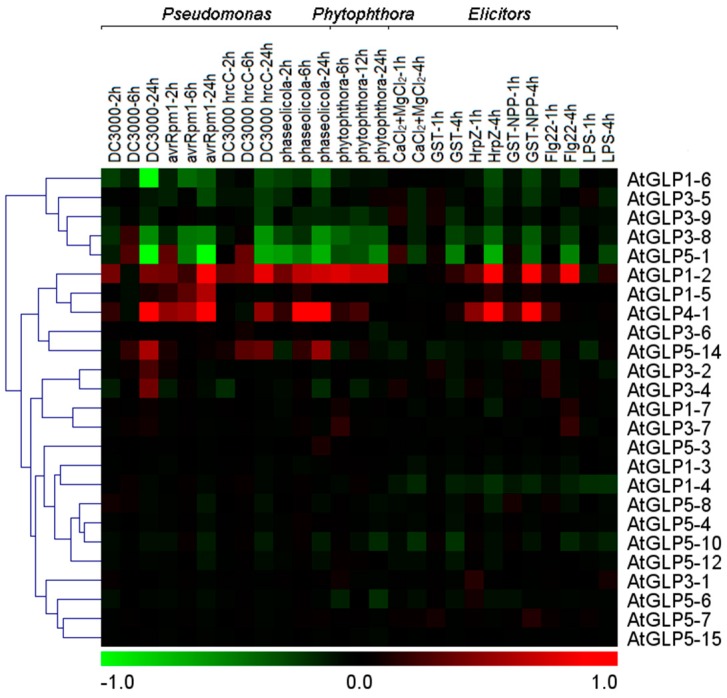
Differential expressions of Arabidopsis *GLP* genes in response to various biotic stress conditions. Heat maps show the log10 fold changes of Arabidopsis *GLP* gene expressions in leaf tissues under different biotic stress conditions such as *Pseudomonas*, *Phytophthora*, and elicitors. Microarray data of Arabidopsis leaves (Table S9) was obtained for different pathogens or elicitors and analysed with respect to the control samples. The colour scale for log10 fold change values are represented by the colour bar shown at the bottom of the heat map; the green colour represents downregulation, black signifies no change in expression, and red shows upregulation.

*AtGLP* genes that respond to pathogens (*Pseudomonas* and *Phytophthora*) and elicitors [flagellin frgment 22 (Flg22), lipopolysaccharides (LPS), harpin (HrpZ), glutathione S-transferases (GST) and GST-necrosis-inducing phytophthora protein 1 (GST-NPP1)] were analysed using microarray data [48,49,50,51]. The expressions of 8 *AtGLP* genes were significantly changed under various biotic stress conditions (Figure 7, Table S9). Among them, *AtGLP3-8* and *AtGLP5-1* were significantly down-regulated under both pathogen and elicitor treatments, while *AtGLP1-2* and *AtGLP4-1* were significantly up-regulated. No disease resistance QTL region was found in Arabidopsis.

The influence of plant defence by GLPs is likely related to their SOD activity [23,27,52]. Superoxide produced by NADPH oxidase or peroxidases in response to pathogen attack is predicted to be dis-mutated to H_2_O_2_ by the GLPs, accounting for the accumulation of H_2_O_2_ [41]. H_2_O_2_ is an important component of plant defence responses, such as cell wall structure protein stiffening and lignification, as well as papillae formation [53]. In previous studies, *GLP* genes located on chromosome 8 in rice were reported to be the key disease-resistance genes [18,25]. Their orthologous *GLP* members in barley and grapevine are also implicated in basal defence responses [23,27], which suggests that the resistance conferred by the *OsGLP* genes on chromosome 8 is via a broad-spectrum, basal mechanism conserved among the Gramineae. Natural selection may have preserved a cluster of *OsGLP* genes on chromosome 8 to provide a stepwise, flexible defence response to pathogen invasion. 

### 2.7. cis-Regulatory Elements in the Promoter of GLP Genes

**Figure 8:**
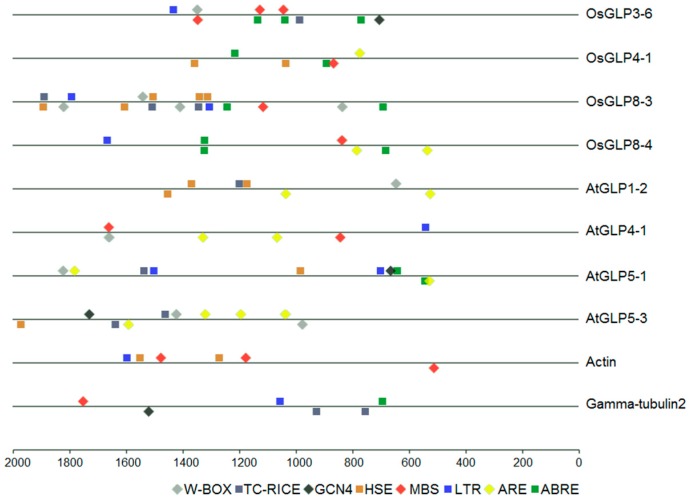
Promoter analysis of eight stress-induced *GLP* genes and two housekeeping genes (Actin and Gamma-tubulin2). Stress-related *cis-*elements of the −2 Kb 5′ upstream region of ten genes are shown. *cis*-Elements in the sense-strand are indicated above the line, and those in the complementary-strand are below the line.

Conserved regulatory elements in *GLP* promoter sequences have been reported to be responsive to environmental stresses and growth factors [21,54]. Here, the promoter analysis was performed for *GLP* genes whose promoter sequences (–2.0 kb) were available in the RGAP and TAIR genome database (Table S10). Eight abiotic/biotic stress-induced *GLP* genes (four from rice and four from Arabidopsis) were randomly selected to compare with sets of two housekeeping promoters (Actin and γ-tubulin2) (Figure 8). A total of eight stress and developmental-related *cis*-elements were selected for promoter analysis, including ABA responsive element (ABRE), anaerobic response element (ARE), low temperature responsive element (LTR), myb-binding site (MBS), heat shock element (HSE), endosperm expression (GCN4), TC-rich repeat responsible for defence, and stress (TC-RICH) and wounding and pathogen response (W-BOX). We found that each *GLP* gene examined in the analysis contained at least four regulatory elements in their promoter regions, while only three regulatory elements were found in Actin promoter and five were found in γ-tubulin2 promoter (Figure 8). Compared with the two housekeeping promoters, two stress-related *cis*-elements (W-BOX and ARE) were only found among the promoter regions of *GLP* genes, and each *GLP* gene contained at least one of the two *cis*-elements. Six regulatory elements were found in the 2 kb upstream region of *OsGLP3-6* which was commonly up-regulated during cold, drought, salt, and different heavy metal stresses, while *AtGLP5-1,* which contained seven regulatory elements, was down-regulated in both *Pseudomonas* and *Phytophthora* biotic stress. Although the promoter regions of *OsGLP8-3* and *OsGLP8-4* contained many stress-related *cis*-elements, both genes were down-regulated under abiotic and biotic stress. The *cis*-element GCN4, which is essential for endosperm-specific expression, was identified in three genes (*OsGLP3-6*, *AtGLP5-1*, and *AtGLP5-3*). These three genes were highly expressed under developmental conditions. Besides, TC-RICH *cis*-element has been identified in the promoter region of five *GLP* genes, suggesting that they might play important roles in response to stress conditions (Figure 8).

### 2.8. Subcellular Localisation and Enzyme Activity of OsGLPs

**Figure 9:**
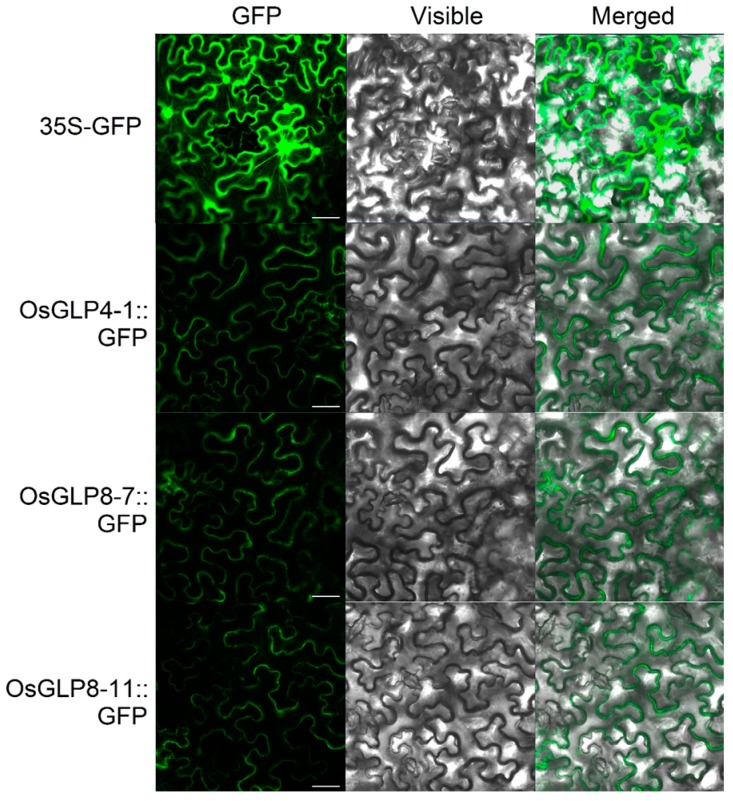
Subcellular localisation of OsGLPs::GFP in *Nicotiana benthamiana* via *Agrobacterium tumefaciens* transformation. Agrobacterium-infiltrated *N. benthamiana* leaves expressing the OsGLPs::GFP and 35S–GFP fusion proteins driven by the CaMV35S promoter. Green fluorescent protein (GFP) fluorescence and differential interference contrast images and visible/GFP merged images are shown from left to right. Scale bar = 25 μm.

**Figure 10:**
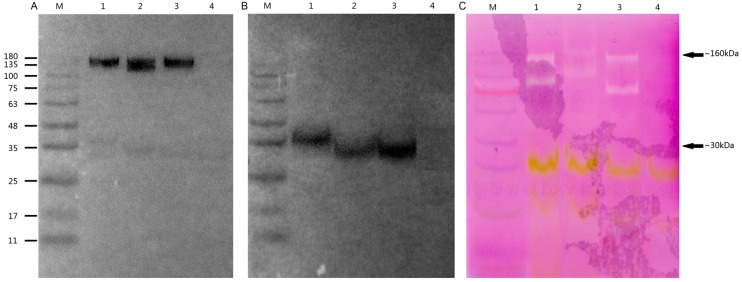
Biochemical characterization of the OsGLP::FLAG proteins production in *Nicotiana benthamiana*. Lane 1, OsGLP8-7::FLAG; Lane 2, OsGLP4-1::FLAG; Lane 3, OsGLP8-11::FLAG; Lane 4, negative control (proteins extracted from wild type *N. benthamiana*); M, molecular size marker. (**A**) Immunodetection of recombinant proteins using FLAG-specific antibody in total protein extracts. Samples were loaded without prior boiling; (**B**) Protein extracts separated as in (**A**) were loaded after boiling; (**C**) Protein extracts separated as in (**A**) were assayed for SOD activity.

Considering *OsGLP* genes on chromosome 8 and 4 were sensitive to various stresses, two genes on chromosome 8 (*OsGLP8-7* and *OsGLP8-11*) and one gene on chromosome 4 (*OsGLP4-1*) were randomly selected to study their subcellular localisations and enzyme activity. Fusion proteins of *OsGLP4-1*, *OsGLP8-7*, and *OsGLP8-11* with C-terminal green fluorescent proteins (GFP) or a triple FLAG tag (3× FLAG) were detected by transient expression in the heterologous plant, *Nicotiana benthamiana*. The results showed that the control vector (35S–GFP) was distributed throughout the whole cell including cell nucleus, cytoplasm and plasma membrane, and cell wall, while the fusion proteins of *OsGLP4-1*, *OsGLP8-7*, and *OsGLP8-11* were only distributed in the cell wall (Figure 9, Figure S1). To further confirm this result, the target *N. benthamiana* was plasmolysed, and no fluorescence remained in the cell plasmalemma. Meanwhile, the three fusion proteins of OsGLP::FLAG were detected in vitro immunoblotting and through SOD activity analysis (Figure 10). These OsGLP fusion proteins showed a band ~160 kDa (without boiling) (Figure 10A) or ~30 kDa (boiling) (Figure 10B). In the SOD activity assay, all these three proteins showed SOD activity. A cell-wall-associated GLP in rice was found to be enriched in sub-epidermal cells [17]. Both gene silencing in epidermal cells of *N. attenuata* and transgenic ectopic expression studies in soybean indicated that the expression of *GLP* genes in the cell wall is related to abiotic and biotic stress [55,56]. *GLP* gene expression has been shown to increase within plant cells during plant interactions with pathogenic microflora, inducing an “oxidative burst” response [24]. GLPs could transform superoxide to H_2_O_2_ and CO_2_, as well as reinforce the cell wall through protein coupling and glycosylation [10]. 

## 3. Materials and Methods

### 3.1. Identification of GLPs in Arabidopsis and Rice

To identify GLPs in Arabidopsis and rice, a hidden Markov model (HMM) search was performed using the TAIR 10 (http://arabidopsis.org) and RGAP 7 (http://rice.plantbiology.msu.edu/) databases (*E*-value ≤ 1 × 10^−10^). All putative GLPs identified were subjected to Pfam (http://Pfam.sanger.ac.uk/) to verify the presence of cupin_1 domain (PF00190). The putative GLPs containing a cupin_1 domain were furtherly confirmed by SMART (http://smart.embl-heidelberg.de/).

### 3.2. Chromosomal Organisation and Phylogenetic Analysis

Chromosome localizations of *GLP* genes were analysed by the MapInspect software [57]. The *GLP* genes separated by a maximum of five genes were identified as tandem duplicated genes. Multiple sequence alignment was performed using MAFFT [58]. Phylogenetic analyses were conducted using Bayesian inference (BI) implemented in MrBayes [59] through the server at CIPRES Science Gateway (http://www.phylo.org/). Four independent runs were performed with four differentially heated Metropolis-coupled Monte Carlo Markov chains for 2 × 10^6^ million generations starting from a random tree, and model parameters were estimated during the analysis. After that, 100 trees were sampled from each run to determine the final consensus tree and posterior probabilities for each clade. The gene structure schematic diagrams were drawn using GSDS (Gene Structure Display Server; http://gsds1.cbi.pku.edu.cn/).

### 3.3. Promoter Analysis

To identify the various *cis*-acting regulatory elements in promoters of *GLP* genes, 2000 base pairs upstream of the CDS were extracted from TAIR 10 and RGAP 7 databases. The upstream sequence was subsequently scanned in the PLACE software [60] to analyse the presence of various *cis*-regulatory elements.

### 3.4. Expression Analysis Using the MPSS and Microarray Data

Expression profiles were obtained from the Arabidopsis and rice MPSS project websites (http://mpss.udel.edu/). MPSS data from 22 mRNA libraries representing 18 different tissues/organs of rice, and 17 mRNA libraries representing 9 different tissue/organs of Arabidopsis, were used for the analysis. The expression profile of Arabidopsis *GLP* genes from microarray data was analysed by AtGenExpress [61]. The samples for these microarray experiments included root tissues under abiotic stress conditions (salt, drought, osmotic, cold, heat, oxidative, genotoxic, wounding, and UV/B), leaf tissues under biotic stress conditions (challenging by *Pseudomonas* and *Phytophthora*), and tissues/organs with different developmental stages [61]. Fold changes at the transcript level were calculated by comparing with controls. For the developmental stage data of Arabidopsis, heat maps were generated based on log10 transformed Affymetrix values, and hierarchical clustering analysis was performed using the MeV software package [62]. For rice *GLP* genes, the expression profiles from Affymetrix GeneChip rice genome arrays [63] were used, including GSE6893 (various stages of development), GSE7951 (stigma and ovary of rice), GSE6901 (rice seedling under cold, salt, or drought stress conditions), GSE25206 (root tissue of rice under heavy metals stress conditions), GSE7256 (seedling infected by *M. grisea*), and GSE10373 (root tissue of two rice cultivars infected by *S. hermonthica*). Among them, the developmental stages of GSE6893 covered root of 7-day old seedling, mature leaf (collected before pollination), young leaf, shoot apical meristem (SD-7-day old seedling), six stages of panicle (i.e., P1 (0–3 cm panicle), P2 (3–5 cm panicle), P3 (5–10 cm panicle), P4 (10–15 cm panicle), P5 (15–22 cm panicle), and P6 (22–30 cm panicle)), and five stages of seed tagged from day of pollination (DAP) (i.e., S1 (0–2 DAP), S2 (3–4 DAP), S3 (5–10 DAP), S4 (11–20 DAP), and S5 (21–29 DAP)). The expression profiles of rice were graphically presented in a heat map based on log2 fold change after value normalisation using R software (R Development Core Team, Vienna, Austria).

### 3.5. Plant Material and Stress Treatment for qRT-PCR Analysis

Seeds of rice cultivar (*O. sativa* “Nipponbare”) were germinated in the hydroponic system. The seedlings were grown in Yoshida medium [64] in a growth chamber at 28 ± 1 °C under a 16-h light/8-h dark photoperiod. After 7 days, various stress treatments were administered to seedlings *viz*. salinity stress (200 mM NaCl for 3 h), dehydration (dried between folds of tissue paper for 3 h), cold stress (4 °C for 3 h), or heavy metal stress (100 µM Cr^6+^/100 µM As^5+^/100 µM Cd^2+^/100 µM Pb^2+^ solutions for 24 h). After treatment, seedlings were cut, weighed, and frozen in liquid nitrogen until further use. Total RNA was extracted using the RNA simple Total RNA Kit (Tiangen Biotech, Beijing, China) according to the manufacturer’s instructions, treated with DNase I, and then converted to cDNA using PrimeScript RT Master Mix (TaKaRa, Dalian, China). To verify the expression pattern of *GLP* genes obtained from microarray data, qRT-PCR was performed using randomly selected genes from *OsGLP* genes sensitive to abiotic stresses. Same tissues were used in qRT-PCR and microarray analysis. Primers were designed in the 3’UTR unique regions of each selected genes using Primer3 software (Tables S11–S13). Each primer was checked for its specificity using BLAST. Three biological replicates of each sample were used for qRT-PCR analysis. To normalise variance among samples, actin was used as the endogenous control. The relative gene expression levels of each target gene were calculated using the ΔΔ*C*_t_ method [65].

### 3.6. Gene Cloning of OsGLPs and Transient Expression

The open reading frame region (without stop codon) of *OsGLP4-1*, *OsGLP8-7*, and *OsGLP8-11* was amplified using designed primers (Tables S12 and S13). PCR products were then cloned into the PHB-GFP or 1306-3FLAG vector using the ClonExpress one-step cloning kit (Vazyme, Piscataway, NJ, USA) to obtain OsGLP::GFP or 1306-3FLAG fusion vector with the CaMV35S promoter. Then, these OsGLP::GFP or OsGLP::FLAG fusion vectors were transformed into *Agrobacterium tumefaciens* strain GV3101 by the freeze thaw method [66]. Transformed *A. tumefaciens* strains were cultivated overnight in 20 mL cultures at 25 °C. Then the cultures was diluted (OD_600_ = 0.5) and infiltrated into leaves of *N. benthamiana* with a 1 mL needleless syringe. 

### 3.7. Subcellular Localization of OsGLPS

After 3 days post-inoculation, OsGLP::GFP fusion proteins in *N. benthamiana* leaves mentioned above were imaged using confocal microscopy (LSM5Pascal; Carl Zeiss, Wetzlar, Germany). Leaves were plasmolysed by incubation in 30% sucrose for 1 h. Leaf sections were mounted on microscope slides in plasmolysis solution. 

### 3.8. Biochemical Analysis in vitro OsGLPs

The OsGLP::FLAG fusion proteins were extracted from ~1 g of *N. benthamiana* leaves [10]. An in-gel SOD activity assay was performed according to the method of Beauchamp and Fridovich [67]. For immune detection, the fusion proteins were detected essentially as described in Rietz et al. [10].

## 4. Conclusions

The present study examined the *GLP* gene family in rice and Arabidopsis. We provided an updated annotation and nomenclature for the *GLP* family and identified several developmental stage-specific, abiotic and biotic stress-responsive *GLP* genes. Tandem duplication of *GLP* genes and the presence of different stress-related *cis*-regulatory elements in the promoter were also analysed. In addition, SOD enzyme activity and subcellular location analysis indicated that the random selected *OsGLP* genes on chromosome 8 and 4 of rice were expressed in the cell wall with SOD activity. It is worth mentioning that evidence of altered expression of *GLP* genes in response to stress is not enough to claim their role for stress tolerance which may be just an indirect consequence of the stress. Thus, more experiments such as studying the stress tolerance of *GLP* mutants are necessary to explore their role in response to stress.

## Supplementary Materials

Supplementary materials can be found at www.mdpi.com/1422-0067/17/10/1622/s1.

## Abbreviations

GLPs	germin-like proteins
SOD	superoxide dismutase
OXO	oxalate oxidase
SDS	sodium dodecyl sulfonate
ROS	reactive oxygen species
MPSS	massively parallel signature sequencing
GFP	green fluorescent protein
BI	bayesian inference
GSDS	gene structure display server
