# Supplementary material for: Genome-Wide Characterization and Expression Analysis of the Germin-Like Protein Family in Rice and Arabidopsis
Source: Int J Mol Sci. 2016 Sep 23;17(10):1622. doi: 10.3390/ijms17101622 (PMC5085655; doi:10.3390/ijms17101622)
Supplement: Supplementary file 1 [file ijms-17-01622-s001.zip › ijms-141989-Supplementary Materials/ijms-141989-supplementary.pdf]

Supplementary Materials: Genome-Wide Characterization and Expression Analysis of the Germin-Like Protein Family in Rice and Arabidopsis

Lu Li, Xihui Xu, Chen Chen and Zhenguo Shen

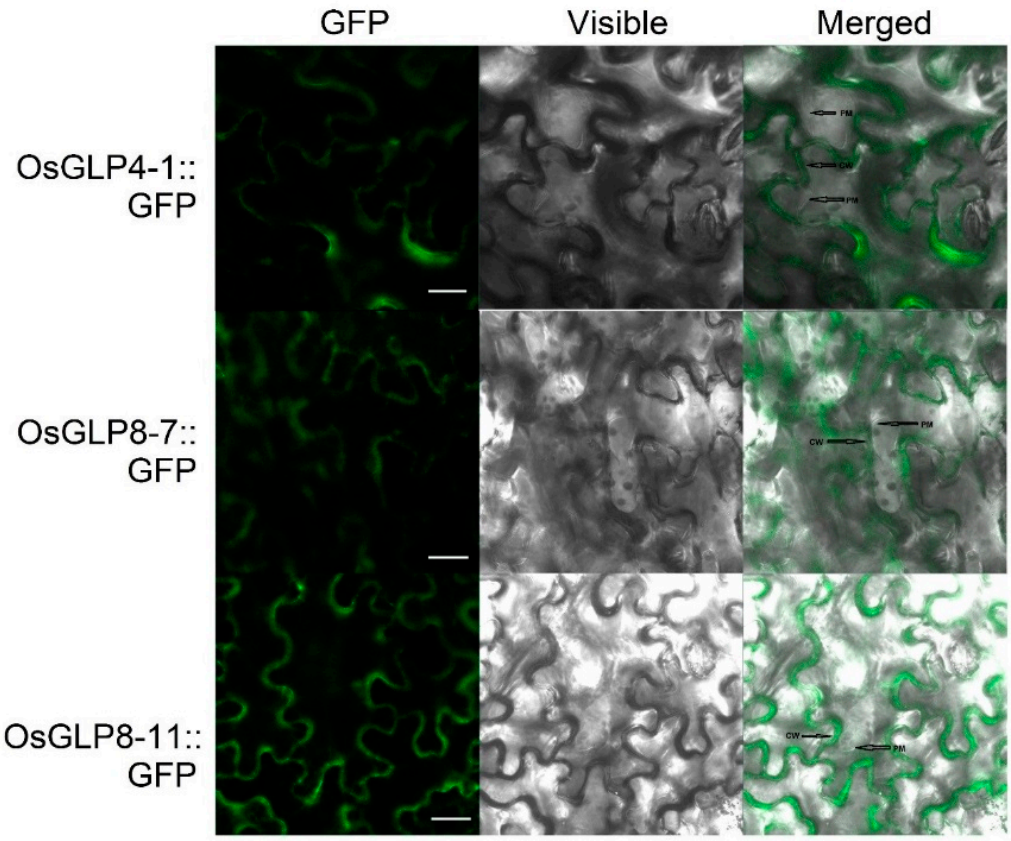

Figure S1. Plasmolysis of *Nicotiana benthamiana* cells expressing OsGLP4-1::GFP, OsGLP8-7::GFP, and OsGLP8-11::GFP. After plasmolysis, GFP fluorescence was detected in the cell wall. All images are from confocal scans performed with a confocal laser scanning microscope. Arrowheads point to the plasma membrane (PM) and cell walls (CW); Scale bar = 25 μm.
